# Frequent Detection of Human Adenovirus from the Lower Gastrointestinal Tract in Men Who Have Sex with Men

**DOI:** 10.1371/journal.pone.0011321

**Published:** 2010-06-25

**Authors:** Marcel E. Curlin, Meei-Li Huang, Xiaoyan Lu, Connie L. Celum, Jorge Sanchez, Stacy Selke, Jared M. Baeten, Richard A. Zuckerman, Dean D. Erdman, Lawrence Corey

**Affiliations:** 1 Fred Hutchinson Cancer Research Center, Seattle, Washington, United States of America; 2 Department of Medicine, University of Washington, Seattle, Washington, United States of America; 3 Department of Laboratory Medicine, University of Washington, Seattle, Washington, United States of America; 4 Department of Epidemiology, University of Washington, Seattle, Washington, United States of America; 5 Department of Global Health, University of Washington, Seattle, Washington, United States of America; 6 Asociación Civil Impacta Salud y Educación, Lima, Peru; 7 Division of Infectious Diseases, Dartmouth-Hitchcock Medical Center, Lebanon, New Hampshire, United States of America; 8 Centers for Disease Control and Prevention, Atlanta, Georgia, United States of America; University of Sao Paulo, Brazil

## Abstract

**Background:**

The association between baseline seropositivity to human adenovirus (HAdV) type 5 and increased HIV acquisition in the Step HIV Vaccine Study has raised questions concerning frequency of acquired and/or persistent Adenovirus infections among adults at high risk of HIV-1 infection.

**Methodology:**

To evaluate the frequency and pattern of HAdV shedding from the lower GI tract, we retrospectively tested rectal swabs for HAdVs in a cohort of 20 HSV-2 positive HIV-positive Peruvian men who have sex with men (MSM) undergoing rectal swabbing three times/week for 18 consecutive weeks, in a prospective study of HSV-2 suppression in HIV infection. Viral DNA was extracted and amplified using a sensitive multiplex PCR assay that detects all currently recognized HAdV types. Molecular typing of viruses was performed on selected samples by hexon gene sequencing. Baseline neutralizing antibody titers to HAdVs −5, −26, −35 and −48 were also assessed.

**Principal Findings:**

15/20 individuals had HAdV detected during follow up. The median frequency of HAdV detection was 30% of samples (range 2.0% to 64.7%). HAdV shedding typically occurred on consecutive days in clustered episodes lasting a median of 4 days (range 1 to 9 days) separated by periods without shedding, suggesting frequent new infections or reactivation of latent infections over time. 8 of the 15 shedders had more than one type detected in follow-up. 20 HAdV types from species B, C, and D were identified, including HAdV-5, −26 and −48, HAdV types under development as potential vaccine candidates. 14/20 subjects were seropositive for HAdV-5; 15/20 for HAdV-26; 3/20 for HAdV-35; and 2/20 for HAdV-48. HAdV shedding did not correlate with CD4 count, plasma HIV-1 viral load, or titers to HAdV-5 or HAdV-35. The sole individual with HAdV-5 shedding was HAdV-5 seropositive.

**Conclusions:**

HAdV shedding was highly prevalent and diverse, including types presently under consideration as HIV vaccine vectors. Subclinical HAdV infection of the GI tract is common among MSM in Peru; the prevalence of HAdV in the enteric tract should be evaluated in other populations. The association between ongoing recent enteric HAdV and the immune response to recombinant HAdV vaccines should be evaluated.

## Introduction

Human adenoviruses (HAdVs) are non-enveloped, moderate-sized DNA viruses that cause asymptomatic infection and clinical syndromes, including upper respiratory tract infection, conjunctivitis, gastroenteritis, infections of the urinary tract, and serious systemic infections in immunocompromised individuals [1], [2]. HAdVs are grouped into seven species, A–G, and further subdivided into at least 52 known types. HAdV shedding from the lower GI tract may occur in healthy adults [3], [4], [5], but is more common in children (where it may represent a primary infection) [6], [7], [8], [9], immunocompromised individuals [2], and persons with HIV infection, particularly those with symptomatic infection and low CD4 counts [3], [10], [11], [12]. In HIV-infected individuals, HAdV shedding has been associated with lower gastrointestinal symptoms such as diarrhea and proctitis in some [3], [13], [14] but not all studies [5], [10], [12]. However, in many cases other copathogens are also present, and it remains unclear to what extent HAdVs other than HAdV-40/41 cause symptomatic infection in the lower GI tract.

HAdVs have been widely explored as vector systems for the delivery of therapeutic and immunogenic gene products because of their ability to infect a range of mammalian host cells, their capacity to accommodate substantial genetic inserts, and their generally favorable safety profile. HAdV type 5 (HAd-5) has been most extensively investigated for applications in gene therapy. In the past ten years, recombinant HAdVs, including type 5, have also been used as vectors in the development of vaccines against several human pathogens, including human immunodeficiency virus (HIV)[15], malaria [16], influenza [17], tuberculosis [18], and Ebola virus [19].

Recent studies have indicated that pre-existing humoral immunity to HAdV-5 is associated with reduced immunogenicity to vaccination with recombinant Ad5 vaccines. In the Step Vaccine Study, a phase II test-of-concept study trial of the Merck HIV-1 gag/pol/nef vaccine, men who have sex with men (MSM) with previous exposure to HAdV-5 through natural infection appear to have had at least transiently increased risk of acquiring HIV after vaccination [15], [20]. The mechanism(s) underlying this observation remain unknown, and may include a spurious association because of the small sample size of the trial, or true biological phenomena such as upregulation of HIV-susceptible target cells within mucosal tissues, and interactions between HAdV-5 immune complexes and dendritic cells resulting in enhanced T cell infection [21]. Recent studies also have demonstrated cross-reactive T cell responses to many human adenoviruses [22] (and unpublished data). These observations draw attention to the potentially important role of natural adenovirus infections in influencing responses to recombinant Ad vector vaccines, and highlight the need to more clearly define the epidemiology and virology of natural human adenovirus infections.

Several HAdV types, including HAdV-5, −26, −35 and −48 are presently under active investigation for use as novel HIV, malaria and TB vaccine vectors [16], [23], [24], [25], [26]. To evaluate whether natural HAdV infections are common in MSM at risk for HIV infection, we examined patterns of HAdV shedding from the genital and lower gastrointestinal tract and tested for preexisting neutralizing antibodies to several proposed HAdV vaccine vectors among HIV-infected MSM undergoing intensive longitudinal mucosal sampling.

## Methods

### Ethics Statement

The human experimentation guidelines of the US Department of Health and Human Services and the individual institutions were followed in the conduct of the clinical research. The institutional review boards of the University of Washington and the Asociación Civil Impacta Salud y Educación approved the protocol. Participants provided written informed consent and were compensated for travel and related expenses.

### Study Subjects

We retrospectively examined genital and anoscopy swab samples obtained from a cohort study of twenty HIV positive MSM in Lima, Peru participating in a prospective randomized, placebo-controlled cross-over study to determine the effect of HSV-2 suppression with acyclovir (40 orally BID) on plasma HIV levels [27]. Study subjects were greater than 18 years of age, with no previous anti-retroviral history and peripheral CD4 counts >200 cells/uL. This cohort offered several advantages for the study of HAdVs; the lengthy observation period and frequent sampling of both the anal and genital areas permitted sensitive detection of short episodes of subclinical infection, and a determination of whether adenovirus infection of the genital tract was present. Because acyclovir has no known antiviral activity against HAdVs [28], [29], the clinical interventions in this cohort allowed an unconfounded study of the prevalence and diversity of adenoviruses infections over time in each participant.

### Clinical visits and procedures

Study subjects returned to the clinic 3 times per week for a total of 18 weeks. Swabbing of the anal and genital regions for viral pathogens was performed by two methods. Rectal mucosal swabbing was performed via anoscopy by placing a sterile Dacron-tipped plastic applicator (Puritan Medical Products, Guilford, ME) against the rectal mucosa 3 to 4 cm above the squamocolumnar junction and rotating for approximately 20 seconds. Surface swabbing of the anogenital region was performed by rubbing the swab over the glans and full length of the penis, the scrotum, perianal skin and completed by inserting the swab in the anus and rotating once (360 degrees). Rectal and anogenital swabbing was performed at each clinic visit. To characterize viral shedding between visits, anogenital swabbing was also self-performed by participants at home. Applicator tips were placed in vials containing 1.0 ml proteinase K digestion buffer (100 mM KCl, 25 mM EDTA, 10 mM Tris-HCl pH 8.0, 1% IGEPAL) and stored at −20°C until processing by the laboratory. Home swabs were maintained at room temperature until the nearest clinic visit and then stored as above.

### DNA extraction and real-time PCR

DNA was extracted from 200 uL of proteinase K digestion buffer using the QIAamp 96 DNA Blood Kit (Qiagen, Inc., Valencia, CA) according to manufacturer instructions and eluted in a total of 100 uL AE buffer. Ten uL of the extract was used to amplify adenoviral DNA using a multiplex PCR assay previously validated to detect 51 recognized HAdV types [30]. The PCR assays were performed on blinded samples at the University of Washington Clinical Virology Laboratories.

### HAdV typing

An aliquot of DNA from all episodes of HAdV was designated for molecular typing based on nested PCR amplification and sequencing of a partial region of the HAdV hexon gene [31], [32]; 116 such samples were submitted to the Centers for Disease Control and Prevention laboratory performing these assays. Predicted amino acid sequences of the hexon hypervariable regions 1–6 (corresponding to amino acid position 192–332 of HAdV type 1 [accession number AP_000512]) were aligned with HAdV prototype sequences (accession numbers DQ115407- DQ115457) using Clustal X ver. 1.83 implemented in BioEdit ver. 7.0.9.0 [33]. Phylogenetic trees were constructed using neighbor-joining method in MEGA version 4.0.2 [34] and midpoint-rooted using minimum F-value optimization. Evolutionary distances were computed using the JTT matrix-based method with all positions containing alignment gaps and missing data eliminated in pair-wise sequence comparisons. For the purpose of this study, HAdVs were typed based on the reference prototype strain that showed the highest sequence identity score at ≥90% and phylogenetic association with ≥90% bootstrap support. HAdV sequences with identity scores <90% were classified by species, but not assigned a type [32], [35]. Sequence submission to GenBank pending manuscript acceptance.

### Serum neutralization

Neutralizing antibody titers for prototype HAdVs −5, −26, −35 and −48 were determined using a modified microneutralization assay [36]. Briefly, 50 µl of heat-inactivated serum (56°C for 30 min) was diluted 1∶10 and then serially 2-fold (Eagle's minimal essential medium with 10% fetal calf serum) in 4-fold replicates in a 96-well microtiter plate. Four TCID_50_ units of pretitered virus in 50 µl was added to each well and incubated for 1 h at room temperature after gentle agitation. Fifty µl of A549 cell suspension (400,000 cells/ml) was then added to each well and mixed thoroughly. After incubation for 5 days at 37°C in 5% CO2, the plate was stained with crystal violet. The neutralization titer was defined as the highest serum dilution that fully inhibited 4 TCID_50_ units of virus.

## Results

A total of 969 rectal swabs were obtained (Table 1). HAdV was detected in 218 samples (22.5%), and 15 of 20 (75%) individuals had HAdV detected at one or more timepoints. For 932 of the rectal samples collected, a concomitant anogenital swab was performed, and HAdV was detected in 97/932 (10.4%) of these samples. In 84/97 (86.6%) of the HAdV-positive anogenital swabs, HAdV was also detected in the corresponding rectal swab collected on the same day. Rectal swabs were also positive on 121 of the days in which no HAdV was detected in the anogenital swab. These data suggested that the source of the adenovirus was invariably the enteric tract, and therefore all further analyses were restricted to samples obtained by anoscopy.

**Table 1:** Cohort demographics and clinical data.

Demographic data	N
Median Age (range)	31 (22,44)
Gender–male (percent)	20 (100)
Median numbers of sex partners in the last month (range)	1 (0,10)
Median numbers of sex partners in the last 6 months (range)	4 (1,40)

Clinical data	N
Number of rectal swabs	969
Median number of rectal swabs per person (range)	49 (33,51)
Median number of positive swabs per person (range)	11 (0,33)
Median percent of positive swabs per person (range)	22.8 (0,64.7)
Median per person mean Log10 Ad copies/ml (range)	3.9 (2.5,5.1)
Baseline HIV viral load (range)	4.3 (3.4,5.1)
Median CD4/mm3 at screen (range)	406 (232, 869)
Number of patients who shed adenovirus at least once	15

Demographic, clinical and virologic data obtained from 20 MSM providing rectal swabs over 18 weeks. “Positive swabs” indicate swabs in which adenovirus was detected by real-time PCR. Baseline HIV viral load provided as log_10_ copies/ml plasma on day 1.

**Figure 1:**
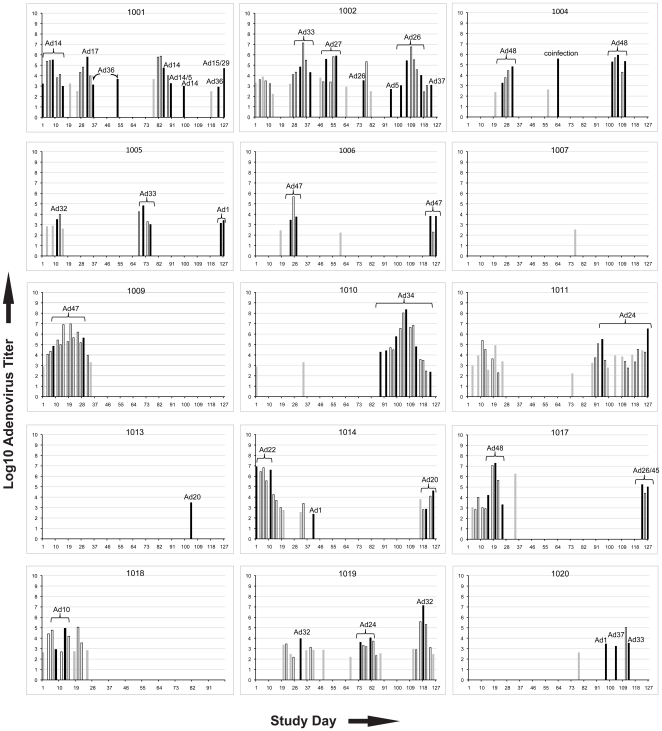
Adenoviral shedding by study day. Pattern of HAdV shedding from the lower gastrointestinal tract by study day. Y axis: Log_10_ HAdV titers from rectal swab material. X-axis: study day. Outlined bars: no typing attempted. Black bars: sequence-based HAdV typing performed. Gray bars: serotyping unsuccessful.

HAdV shedding in the rectal mucosa occurred on 21.1% of days sampled (range 2.6–52.5). Mean log_10_ HAdV concentration was 4.1/ml buffer (range 2.2–8.3). The pattern of HAdV shedding in all 15 persons in whom virus shedding was detected is shown in Figure 1. Enteric infection was characterized by frequent clusters or “bursts” of shedding. HAdV concentrations in rectal swabs varied by episode, but were generally bell-shaped over each “cluster” period. Sequential infections of multiple types were documented in 9 of the 15 shedders. Median CD4 count was 406 cells/µl (range 232–869) and mean plasma HIV-1 level was 4.3 log_10_ copies/ml. CD4 count and plasma HIV-1 load did not differ between participants with and without HAdV shedding (Mann-Whitney, p = 0.349 and p = 0.497, resp.). HAdV shedding patterns did not correlate with shedding of HSV2 or cytomegalovirus (CMV), nor with use of oral acyclovir (data not shown).

**Figure 2:**
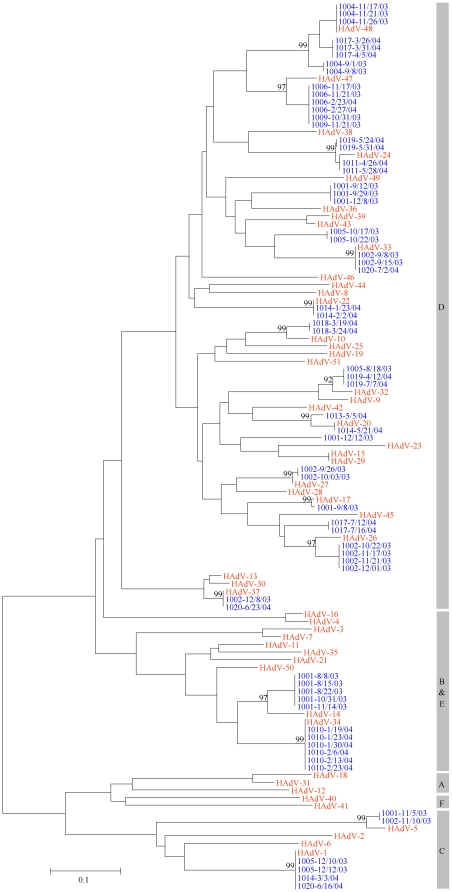
Phylogenetic tree of hexon sequences and reference strains. Neighbor-joining phylogenetic tree of partial length HAdV hexon gene sequences obtained from 64 clinical samples (blue) and reference strains (red).

Molecular typing was attempted on a representative subset of samples from each shedder, selected to cover all observed shedding episodes in this cohort (N = 116 samples). Fifty samples could not be definitively assigned to a known HAdV type due to insufficient amplified DNA for sequencing, the presence of a mixed virus infection that could not be resolved or, in a few cases, to significant differences between the sequenced strain and known reference sequences. Among the 56 positive samples successfully sequenced, numerous HAdV types distributed among species B, C, and D were identified (Table 2; Figure 2). Multiple HAdV types were detected in eight subjects, while only a single type was observed in the remaining seven individuals shedding virus. Four genetically distinct viruses were identified that had <90% predicted amino acid sequence identity with the prototype viruses, and may therefore represent new types. Three unique genetic variants of HAdV-48 were identified in two study subjects; subject 1004 shed one variant in September followed by a second variant in November, 2003 (Figure 2). The most prevalent types were HAdV-1 and −33, which were seen in three individuals. The remaining types were each seen in one or two individuals among the 15 shedders.

**Table 2:** Hexon sequence identity with nearest reference strain.

Patient ID	Study Day	Best matching	% aa identity	2^nd^ best match	% aa identity
		Ref. strain	score	Ref. Strain	score
1001	8/8/03	Ad14	91.4	Ad50	84.3
1001	8/15/03	Ad14	91.4	Ad50	84.3
1001	8/22/03	Ad14	91.4	Ad50	84.3
1001	9/8/03	Ad17	99.3	Ad26	81.6
1001	9/12/03	(Ad36)	81.8	Ad33	76.2
1001	9/29/03	(Ad36)	81.8	Ad33	76.2
1001	10/31/03	Ad14	91.4	Ad50	84.3
1001	11/5/03	Ad5	95.6	Ad6	62.5
1001	11/14/03	Ad14	91.4	Ad50	84.3
1001	12/8/03	(Ad36)	81.8	Ad33	76.2
1001	12/12/03	(Ad15/Ad29)	80.6	Ad17	80.2
1002	9/8/03	Ad33	100	Ad43	81.1
1002	9/15/03	Ad33	100	Ad43	81.1
1002	9/26/03	Ad27	99.2	Ad28	83.9
1002	10/3/03	Ad27	99.2	Ad28	83.9
1002	10/22/03	Ad26	92.5	Ad17	81.5
1002	11/10/03	Ad5	95.6	Ad6	62.5
1002	11/17/03	Ad26	92.5	Ad17	81.5
1002	11/21/03	Ad26	92.5	Ad17	81.5
1002	12/1/03	Ad26	92.5	Ad17	81.5
1002	12/8/03	Ad37	100	Ad13	94.8
1004	9/1/03	Ad48	95.3	Ad47	82.5
1004	9/8/03	Ad48	95.3	Ad47	82.5
1004	11/17/03	Ad48	100	Ad47	84.5
1004	11/21/03	Ad48	100	Ad47	84.5
1004	11/26/03	Ad48	100	Ad47	84.5
1005	8/18/03	Ad32	95.8	Ad9	72.3
1005	10/17/03	(Ad33)	83.9	Ad36	81.8
1005	10/22/03	(Ad33)	83.9	Ad36	81.8
1005	12/10/03	Ad1	100	Ad6	71.5
1005	12/12/03	Ad1	100	Ad6	71.5
1006	11/17/03	Ad47	90.6	Ad38	77.9
1006	11/21/03	Ad47	90.6	Ad38	77.9
1006	2/23/04	Ad47	90.6	Ad38	77.9
1006	2/27/04	Ad47	90.6	Ad38	77.9
1009	10/31/03	Ad47	90.6	Ad38	77.9
1009	11/21/03	Ad47	90.6	Ad38	77.9
1010	1/19/04	Ad34	100	Ad14	81.9
1010	1/23/04	Ad34	100	Ad14	81.9
1010	1/30/04	Ad34	100	Ad14	81.9
1010	2/6/04	Ad34	100	Ad14	81.9
1010	2/13/04	Ad34	100	Ad14	81.9
1010	2/23/04	Ad34	100	Ad14	81.9
1011	4/26/04	Ad24	97.9	Ad38	80.4
1011	5/28/04	Ad24	97.9	Ad38	80.4
1013	5/5/04	Ad20	95.1	Ad42	80.4
1014	1/23/04	Ad22	100	Ad48	73.8
1014	2/2/04	Ad22	100	Ad48	73.8
1014	3/3/04	Ad1	100	Ad6	71.5
1014	5/21/04	Ad20	100	Ad42	79.7
1017	3/26/04	Ad48	95.9	Ad47	82.5
1017	3/31/04	Ad48	95.9	Ad47	82.5
1017	4/5/04	Ad48	95.9	Ad47	82.5
1017	7/12/04	(Ad26)	87.7	Ad45	83.7
1017	7/16/04	(Ad26)	87.7	Ad45	83.7
1018	3/19/04	Ad10	93.2	Ad25	81.2
1018	3/24/04	Ad10	93.2	Ad25	81.2
1019	4/12/04	Ad32	95.8	Ad9	72.3
1019	5/24/04	Ad24	97.2	Ad38	80.4
1019	5/31/04	Ad24	97.2	Ad38	80.4
1019	7/7/04	Ad32	95.8	Ad9	72.3
1020	6/16/04	Ad1	100	Ad6	71.5
1020	6/23/04	Ad37	100	Ad13	94.8
1020	7/2/04	Ad33	100	Ad39/Ad43	81.1

Predicted partial-length hexon amino acid sequence identity with nearest and second nearest reference sequences, by subject ID and sample collection day for all samples with available sequence data. Sequence relatedness to reference strains is calculated based the percentage of shared amino acids at each position in an alignment of sequences. Samples with no closely matching reference strain (< 90% sequence identity to known adenoviral types) are denoted by “()”, indicating possible new viral types.

Baseline serum neutralizing antibodies to HAdV-5, −26, −35 and −48 were assessed in all study participants. Fourteen of 20 individuals (70%) were seropositive for HAdV-5; 15 of 20 (75%) for HAdV-26; three of 20 (15%) for HAdV-35; and 2 of 20 (10%) for HAdV-48 (Table 3).

**Table 3:** Baseline serum neutralizing antibody titers by subject.

Subject ID	Ad5 titer	Ad26 titer	Ad35 titer	Ad48 titer
1001	>1∶80	>1∶80	Neg	1∶10
1002	>1∶80	Neg	Neg	Neg
1003	1∶80	Neg	Neg	Neg
1004	>1∶80	1∶20	Neg	Neg
1005	Neg	1∶10	Neg	Neg
1006	>1∶80	>1∶80	Neg	Neg
1007	>1∶80	>1∶80	Neg	Neg
1008	Neg	>1∶80	1∶10	Neg
1009	1∶80	1∶10	Neg	Neg
1010	Neg	>1∶80	Neg	Neg
1011	Neg	Neg	Neg	Neg
1012	1∶10	>1∶80	1∶20	>1∶80
1013	1∶20	1∶20	Neg	Neg
1014	Neg	>1∶80	Neg	Neg
1015	1∶40	Neg	Neg	Neg
1016	Neg	Neg	Neg	Neg
1017	1∶20	1∶40	>1∶80	Neg
1018	1∶40	1∶40	Neg	Neg
1019	1∶80	1∶20	Neg	Neg
1020	1∶20	1∶40	Neg	Neg
% positive	70%	75%	15%	10%

Serum neutralizing antibody titers to HAdV-35, −5, −26 and −48 in each subject at beginning of study.

## Discussion

In this study, we examined the prevalence and shedding patterns of HAdV from the lower gastrointestinal tract of HIV-positive Peruvian MSM through intensive longitudinal sampling over 18 weeks. We observed viral shedding in 75% of study participants, who collectively yielded at least 20 different HAdV types including four potentially new types and two additional variants of type 48. Several previous studies have examined the prevalence of enteric viruses in a variety of populations [2], [3], [4], [5], [6], [7], [8], [10], [11], [12], [13], [14], [37], [38], [39], [40], [41], [42], [43], [44]. While HAdV infections in otherwise healthy individuals generally manifest as transient infections with complete resolution, numerous studies have demonstrated the potential for these viruses to establish prolonged and/or latent infection in a variety of sites including adenoidal tissue [45], peripherally circulating mononuclear cells [46], the urinary tract [44], the conjunctiva [47], and the GI tract [9], [11].

One striking difference between this report and prior studies is the remarkably high prevalence of HAdV shedding observed in our study. Earlier work has suggested shedding rates ranging from 0–4% in healthy adults and from 0–28% in selected individuals with HIV infection. In contrast, we observed shedding in 75% of study subjects. This large disparity is likely due to the detection methods employed. Few studies have utilized sensitive molecular methods and the frequent sampling entailed in this protocol; past studies have generally relied on electron microscopy, enzyme immunoassays and cytopathogenicity assays for detection of HAdVs, and employed relatively sparse sampling. In our study, HAdV shedding was episodic, occurring on only 30% of days sampled among those excreting virus at any time. It is therefore likely that many instances of viral shedding were missed in studies relying on limited sampling, due to the short duration of viral shedding episodes. We also note that in this study, we have examined HIV-positive persons who might also manifest more frequent or prolonged HAdV reactivity [11], [48]. Additionally, we observed that the prevalence of positive neutralizing antibody titers to HAdV-5, HAdV-35 and HAdV-48 were consistent with several previously published reports [24], [49], [50], [51]. However, in this cohort, the baseline seroprevalence to HAdV-26 was considerably higher than in most earlier studies, with the exception of one study reporting seroprevalence rates ranging from 12%–88% depending on the geographic region studied [51].

In this study, we have defined several previously underappreciated aspects of enteric HAdV infection, including an extremely high prevalence of viral shedding, a high rate of serological immunity to “rare” serotypes, the frequent occurrence of mixed infections, and several new adenoviral serotypes. The methods used in this study do not permit us to conclusively distinguish between incident infection, persistent infection with intermittent viral reactivation, and passive shedding of viral nucleic acids associated with latently infected cells in the absence of viral replication. However, in several subjects, the presence of discrete shedding episodes with rising and falling viral loads is highly suggestive of active replication. PCR detection of adenovirus from the enteric tract has been shown to correlate with presence of infectious virus in non-human primates and humans [52], and thus the shedding we have observed in our study is likely reflect the presence of infectious virus. Whether intermittent shedding of the same type of HAdV in several subjects over time indicates low-grade persistent infection or episodic reactivation of latent infection is unclear. We also note that individuals 1002, 1004 and 1017 shed viruses for which the corresponding baseline serologies were negative (HAdV-26, −48 and −48, respectively), suggesting that these episodes were new infections. Sequence-based typing of mixed infections is likely to only identify the predominant genotype present, and novel genetic variants are difficult to identify by the methods used. It is therefore likely that the true diversity of HAdV types present has been underestimated in this study.

This study is limited by modest cohort size, relatively narrow geographical sampling and by the limited serological data presently available. However, our serology results suggest that infection with “rare” HAdVs with and/or without persistent shedding may be more prevalent than previously thought [24], [49], [50] among certain HIV+ MSM populations, and highlight the need to further investigate the prevalence and transmission of HAdVs in sexually active MSM and other populations at high risk of acquiring sexually transmitted diseases. It is presently unknown to what extent the presence of natural adenoviral infections may interact with vaccines based on similar strains. These results must therefore also be considered in the context of vaccine development and immunization efforts using adenoviral vaccine vectors.
